# Exploring Genomic, Geographic and Virulence Interactions among Epidemic and Non-Epidemic St. Louis Encephalitis Virus (Flavivirus) Strains

**DOI:** 10.1371/journal.pone.0136316

**Published:** 2015-08-27

**Authors:** Luis A. Diaz, Sandra E. Goñi, Javier A. Iserte, Agustín I. Quaglia, Amber Singh, Christopher H. Logue, Ann M. Powers, Marta S. Contigiani

**Affiliations:** 1 Laboratorio de Arbovirus, Instituto de Virología ‘‘Dr. J. M. Vanella”, Facultad de Ciencias Médicas, Universidad Nacional de Córdoba, Córdoba, Argentina; 2 Instituto de Investigaciones Biológicas y Tecnológicas, CONICET—Universidad Nacional de Córdoba, Córdoba, Argentina; 3 Área de Virosis Emergentes y Zoonóticas, Laboratorio de Ingeniería Genética y Biología Celular y Molecular, Instituto de Microbiología Básica y Aplicada, Departamento de Ciencia y Tecnología, Universidad Nacional de Quilmes, Bernal, Buenos Aires, Argentina; 4 Laboratorio de Bioinformática Estructural, Fundación Instituto Leloir, CABA, Buenos Aires, Argentina; 5 Centers for Diseases Control and Prevention. Fort Collins, Colorado, United States of America; 6 Novel and Dangerous Pathogens Training, Public Health England, Porton Down, United Kingdom; University of Texas Medical Branch, UNITED STATES

## Abstract

*St*. *Louis encephalitis virus* (SLEV) is a re-emerging arbovirus in South America. In 2005, an encephalitis outbreak caused by SLEV was reported in Argentina. The reason for the outbreak remains unknown, but may have been related to virological factors, changes in vectors populations, avian amplifying hosts, and/or environmental conditions. The main goal of this study was to characterize the complete genome of epidemic and non-epidemic SLEV strains from Argentina. Seventeen amino acid changes were detected; ten were non-conservative and located in proteins E, NS1, NS3 and NS5. Phylogenetic analysis showed two major clades based on geography: the North America and northern Central America (NAnCA) clade and the South America and southern Central America (SAsCA) clade. Interestingly, the presence of SAsCA genotype V SLEV strains in the NAnCA clade was reported in California, Florida and Texas, overlapping with known bird migration flyways. This work represents the first step in understanding the molecular mechanisms underlying virulence and biological variation among SLEV strains.

## Introduction

*Saint Louis encephalitis virus* (SLEV) (*Flavivirus*, *Flaviviridae*), a reemerging arbovirus in South America, has been responsible for febrile disease and human encephalitis cases in Argentina in 2002, 2005 and 2010, and in Brazil in 2004 and 2006[1–4]. SLEV first reemerged in the central region of Argentina (Córdoba and Santa Fe Provinces) in 2002, when two cases of encephalitis and three fever cases were reported [5]. The first human epidemic of SLEV outside of North America was reported in 2005, when 47 laboratory-confirmed clinical cases of SLE, including nine fatalities, were reported in Córdoba Province (Argentina)[1].

The geographic distribution of SLEV encompasses tropical, sub-tropical and much of the temperate-tropical zones of the Western Hemisphere which includes most of the populated land masses of North and South America [6]. In the USA, SLEV is naturally maintained by mosquito-to-bird transmission involving several *Culex* (*Cx*.) spp. Mosquitoes and a variety of bird species, including the house sparrow (*Passer domesticus*), house finch (*Haemorhousmexicanus*) and mourning doves (*Zenaidamacroura*) [6].

Most phylogenetic relationships among SLEV strains are based on the complete envelope gene sequence [7]. A total of 8 genotypes are presently recognized (I-VIII)[8]. Genotypes I and II are prevalent in the USA, while the others are traditionally found only in Central and South America[8]. During the 2005 SLEV outbreak in Argentina two genotype III SLEV strains were isolated from *Cx*. *quinquefasciatus* mosquitoes[9]. Moreover, during the reemergence of SLEV in Buenos Aires province, molecular evidence suggested an association with same genotype III[10]. Although not completely understood, evidence supports a multicausal event for SLEV reemergence. Introduction of a novel more virulent strain, an increase of *Cx*. *quinquefasciatus* and *Cx*. *interfor* mosquito vector abundance, and highly susceptible avian host availability are potential explanations[11,12]. A retrospective study shows that no previous activity was documented for this genotype in Cordoba city prior to the 2005 outbreak[11]. Moreover, some biological differences among epidemic (CbaAr-4005, Ep) and non-epidemic (79V-2533, NEp) viral strains were detected. For example, house sparrows inoculated with SLEV CbaAr-4005 Ep developed higher and long lasting viremias[12].

In flaviviruses, several mutations have been associated with phenotype alteration, including those linked to virulence. Unfortunately, the genotypic evidence associated with a virulent SLEV phenotype is lacking. The main objective of this study was to characterize the complete genome of SLEV strains from Argentina, to identify molecular differences among Ep and NEp SLEV strains, and to associate these differences with ecologic, epidemiologic and geographic trends.

## Materials and Methods

### Viral strains

Two SLEV genotype III strains were completely sequenced (79V-2533 and CbaAr-4005). The 79V-2533 strain was isolated from *Culex* (*Culex*) spp. mosquitoes collected in Esperanza city (Santa Fe Province, Argentina) in 1978, during a non-epidemic period. The epidemic strain (CbaAr-4005) was isolated from *Cx*. *quinquefasciatus* mosquitoes collected during the SLEV human encephalitis outbreak of 2005. Viruses were propagated on VERO cell monolayers inoculated with 100μl of CbaAr-4005 or 79V-2533viral strains. Virus was harvested on the 6^th^ day post-infection (dpi) by centrifuging the supernatant after one freeze/thaw cycle.

### RNA extraction, reverse transcription and PCR amplification

Vero cell supernatant was used for RNA viral extraction employing the commercial QIAamp viral RNA MiniSpin Kit (Qiagen). For reverse transcription and PCR, two commercial kits were used, Titan One Tube RT-PCR System (Roche) and Titan One Tube RT-PCR Kit (Roche, Reaction Mix 2), following the manufacturer’s instructions.

### Primer design and genome sequencing

The sequencing strategy and primer design to obtain the complete sequence for CbaAr-4005 and 79V-2533 strains were based on a consensus sequence generated from Kern217 (DQ525916.1 and NC_007580.2) and Argentine66 (AY632544.1).

For the 5′UTR amplification and sequencing, a commercial kit (Ambion #AM1700 First Choice RLM Race) was used. For the 3′UTR, the A-Plus Poly (A) Polymerase Tailing Kit (Epicentre Biotechnologies) was used. The manufacturer instructions were applied with the following exceptions: 2.5μl RNAase inhibitor (40U/μl), and 0.5μl A-Plus Poly A (4U/μl) were added and the reaction incubated at 37°C for 10min. The Titan One Tube RT-PCR Kit (Roche) was used for genomic amplification. For sequencing of the amplified fragments the same protocol as that for the 5′UTR was used.

The partial fragments generated for each strain were analyzed and individually selected for assembly to generate a consensus genome sequence using the SeqMan II (v. 5.03) program provided within the LaserGene (DNAStar) package. The complete genome sequences for both strains were submitted to GenBank with the accession numbers FJ753286.2 and FJ753287.2.

### Multiple alignments and bioinformatics analyses

Multiple alignments of 29 analyzed sequences were generated by ClustalW[13] with the MEGA 6 program (http://www.megasoftware.net). Most of sequences analyzed possessed coding region sequences only, missing the 5′and 3′non coding regions as well as the last portion of NS5 gene. Therefore, for the bioinformatics analysis, all sequences were truncated in order to use the same sequence range.

The coding region was identified using Clone Manager (http://www.scied.com/pr_cmpro.htm) software. The polyprotein cleavage sites were determined following the method described by Rice and Strauss [14]. The SignalP-NN program was employed (http://www.cbs.dtu.dk/services/) for proteolytic sites and protease recognition signals. A comparative study among other flaviviruses was carried out based on previously published findings[15]. In the Ep and NEp comparative analyses, conservative and non conservative amino acid substitutions were identified and classified according to the Dayhoff matrix.

The relative homology (RH) profile was calculated for the nucleotide and amino acid sequences of the 29 ORF sequences analyzed. These profiles reflect the conservation grade between different regions of genome and proteins. The relative homology was calculated by a strategy based in overlapping windows, using the consensus symbols obtained in the ClustalW alignments. The media value was calculated using the addition of the total values divided for the window size and plotted (Ghiringhelli, P.D. and Iserte, J.A., unpublished). For homology calculations between protein sequences, the window size was 11 residues and arbitrary values of +1 for identical residues, +0.5 and +0.25 for conservative changes, and -1 for non identical residues were assigned. The non coding regions were analyzed by Sequence Logos tool (http://weblogo.berkeley.edu/logo.cgi) and the RNA secondary structure was modeled (MFold, http://www.bioinfo.rpi.edu/~zukerm). Briefly, the Sequence Logo is a graphic representation of a multiple alignment of sequences. After the alignments were generated, the sequences were manually curated (using *Dengue 2 virus* (DENV2) strains (NC001474.2: DENV-2/16681 and DQ181806.1: DENV-2/Th2_0038_74) and SLEV CbaAr-4005 and 79V-2533 strains), the 5′UTR and 3′UTR were compared, and the different signals and structures were located [16].

Recombination in SLEV polyprotein open reading frame sequences was tested for with SBP and GARD software on Datamonkey web server [17].

### Phylogenetic analyses

An alignment containing 29 SLEV strains (Table 1) and 3 sequences as the outgroup strains (*West Nile virus*, *Japanese encephalitis virus*, and *Murray Valley Encephalitis virus*) was generated using MEGA6 program (http://www.megasoftware.net/). The substitution model used in the phylogenetic reconstruction was GTR with a proportion of invariant sites and Gamma distributed rates. The substitution model was selected with jModelTest 2.1.5 [18]. Phylogenetic reconstruction was made by Bayesian inference using BEAST 2.0[19]. Site model was partitioned by codon position. Substitution models in Beast were tested and compared with the model selected with jModelTest. Monte Carlo Markov chain (MCMC) length was 50 million, sampling every 1000. The Tracer program (http://tree.bio.ed.ac.uk/software/tracer/) was used to analyze convergence of MCMC. The tree presented is the maximum clade credibility tree summarized from MCMC data with a burn in of 10%. Phylogenetic reconstruction was also made in MEGA6 software using the Maximum Likelihood method, with 1000 replicates for bootstrap analysis.

**Table 1:** St. Louis encephalitis virus strains and their biological features.

SLEV Strain	Geographic Origin	Year of isolation	Host	Rodent Pathogenicity^¥^	Avian Viremia^±^	Accesion Number
79V_2533	Santa Fe, Argentina	1979	*Culex* spp. (Mosquito)	n.d.	n.d.	FJ753287.1
CbaAr_4005	Córdoba, Argentina	2005	*Culex quinquefasciatus* (Mosquito)	High^D^	High^C^	FJ753286.1
BeAr23379	Belem, Brasil	1960	*Sabethes belisarioi* (Mosquito)	Low^A^	Low^B^	EF158048.1
Kern217	California, USA	1989	*Culex tarsalis* (Mosquito)	n.d.	n.d.	DQ525916.1
904.3	Kentucky, USA	1955	*Colaptes auratus* (Bird)	High^A^	High^B^	EF158049.1
MSI_7	Mississippi, USA	1975	*Passer domesticus* (Bird)	High^A^	High^B^	DQ359217.1
GMO94	Guatemala	1969	*Culex nigripalpus* (Mosquito)	High^A^	High^B^	EF158051.1
V2380_42	Texas, USA	2001	*Culex quinquefasciatus* (Mosquito)	n.d.	n.d.	EF158052.1
BeAn246262	Belem, Brazil	1973	*Didelphis marsupialis* (Mammal)	High^A^	High^B^	EF158053.1
75D90	Perú	1975	Mosquito	High^A^	Low^B^	EF158054.1
TBH28	Florida, USA	1962	Human	n.d.	n.d.	EF158055.1
TRVL_9464	Trinidad	1955	*Psorophora ferox* (Mosquito)	Low^A^	Low^B^	EF158056.1
78_A_28	Guatemala	1978	Mosquito	n.d.	n.d.	EF158057.1
65V310	México	1965	*Butorides virescens* (Bird)	Low^A^	High^B^	EF158059.1
GML903797	Panamá	1983	Chickens (Bird)	n.d.	n.d.	EF158060.1
69M_1143	Florida, USA	1969	*Procyon lotor* (Mammal)	Low^A^	Low^B^	EF158061.2
FL79_411	Florida, USA	1979	*Culex nigripalpus* (Mosquito)	n.d.	n.d.	EF158062.1
CorAn9124	Córdoba, Argentina	1966	*Calomys musculinus* (Mammal)	Low^A^	Low^B^	EF158063.1
GML902612	Panamá	1973	*Haemagogus equines* (Mosquito)	n.d.	n.d.	EF158064.1
TNM4_711K	Tennesse, USA	1974	*Culex pipiens* (Mosquito)	High^A^	High^B^	EF158065.1
GHA 3	Florida, USA	1962	Human	High^A^	n.d.	EF158066.1
BeAn247377	Belem, Brasil	1973	*Hylophilax poecilonata* (Bird)	n.d.	n.d.	EF158067.1
CorAn9275	Córdoba, Argentina	1967	*Mus musculus* (Mammal)	Low^A^,^D^	Low	EF158068.1
72V_4749	Colorado, USA	1972	*Culex tarsalis* (Mosquito)	High^A^	Low^B^	EF158069.1
Parton	Missouri, USA	1933	Human	n.d.	n.d.	EF158070.1
Hubbard	Missouri, USA	1937	Human	n.d.	n.d.	EU566860.1
IMP115	California, USA	2003	*Culex tarsalis* (Mosquito)	-	-	JF460774.1
Palenque-A770	Palenque, México	2008	*Culex nigripalpus* (Mosquito)	-	-	JQ957869.1
Palenque-C475	Palenque, México	2008	*Culex nigripalpus* (Mosquito)	-	-	JQ957868.1

^**¥**^ Pathogenicity feature observed in swiss albino mice or monkeys according to cited literature.

^**±**^Viremia observed in subcutaneously inoculated birds (house sparrows/chicks).

^A^[23];

^B^[24];

^C^[12];

^D^[25].

n.d.: No data available.

### Geographic distribution and genetic diversity

The association between geographic and genetic distance of the 29 SLEV strains examined was explored through symmetric classic Procrustes analysis [20]. Genetic and geographic distance matrices were constructed. Geographic coordinates were obtained by Google Earth application and geodesic distances were calculated. Genetic distances were calculated using Kimura 2-parameter as the evolutionary model under MEGA 6 software [21]. Distance matrices were used as input for ordination analysis. Non Metric Multidimensional Scaling (NMDS) of each distance matrix showed the ordination constellation and potential outlier in pairwise fashion. After removal of the outliers, the genotype distance’s NMDS was transposed, rotated and scaled on the geographic distance`s NMDS. The selected statistics metrics were Procrustes’s correlation (*t0*), sum of squared errors (*m* ^*2*^), scaling and rotation. The significance of *t0* was tested against of the distribution pseudo Procrustes`s correlation (*t*) with10,000 permutations. All analysis were performed in R [22] with the Vegan package[20]. The workflow script to run this analysis on R is available in the supplementary data file provided (please see S1 File).

### Association of mutations with virulence and viremia

We used bibliographic data available in order to determine biological features associated with all studied viral strains including: source of isolation (mosquito, bird, mammals), viremia in birds, and virulence in mice[12,23–25]. SLEV strains were classified as high virema (HV) or low viremia (LV), and high pathogenicity (HP) or low pathogenicity (LP) (Table 1). An amino acid change survey was carried out for each protein in order to find non conservative mutations through the ORF.

Multivariate analyses were employed to explore patterns between genomic mutations and biological characteristics. Analysis of 200 point mutations observed in a pool of 14 SLEV strains with respect to the multidimensional distribution of all descriptors (biological features) was carried out. As a consequence of the binary nature of the data (i.e: a Chi-square distance between mutation) a Correspondence Analysis was selected[26]. Once CA analyses were carried out, correlations between source of isolation (birds, mammals, mosquitoes), viremogenic capacity in birds (High and Low) and virulence in mice (High and Low) were tested. Spearman’s correlation indexes and bootstrap significance were calculated using “corrgram” and “psych” packages in R. After multivariate analysis, selected mutations were tested in contingency tables for virulence and viremia using a Fisher’s exact test and ODD ratio. These analyses were carried out with Infostat software package (www.infostat.com.ar).

## Results

### Epidemic vs Non-Epidemic strains

**Fig 1:**
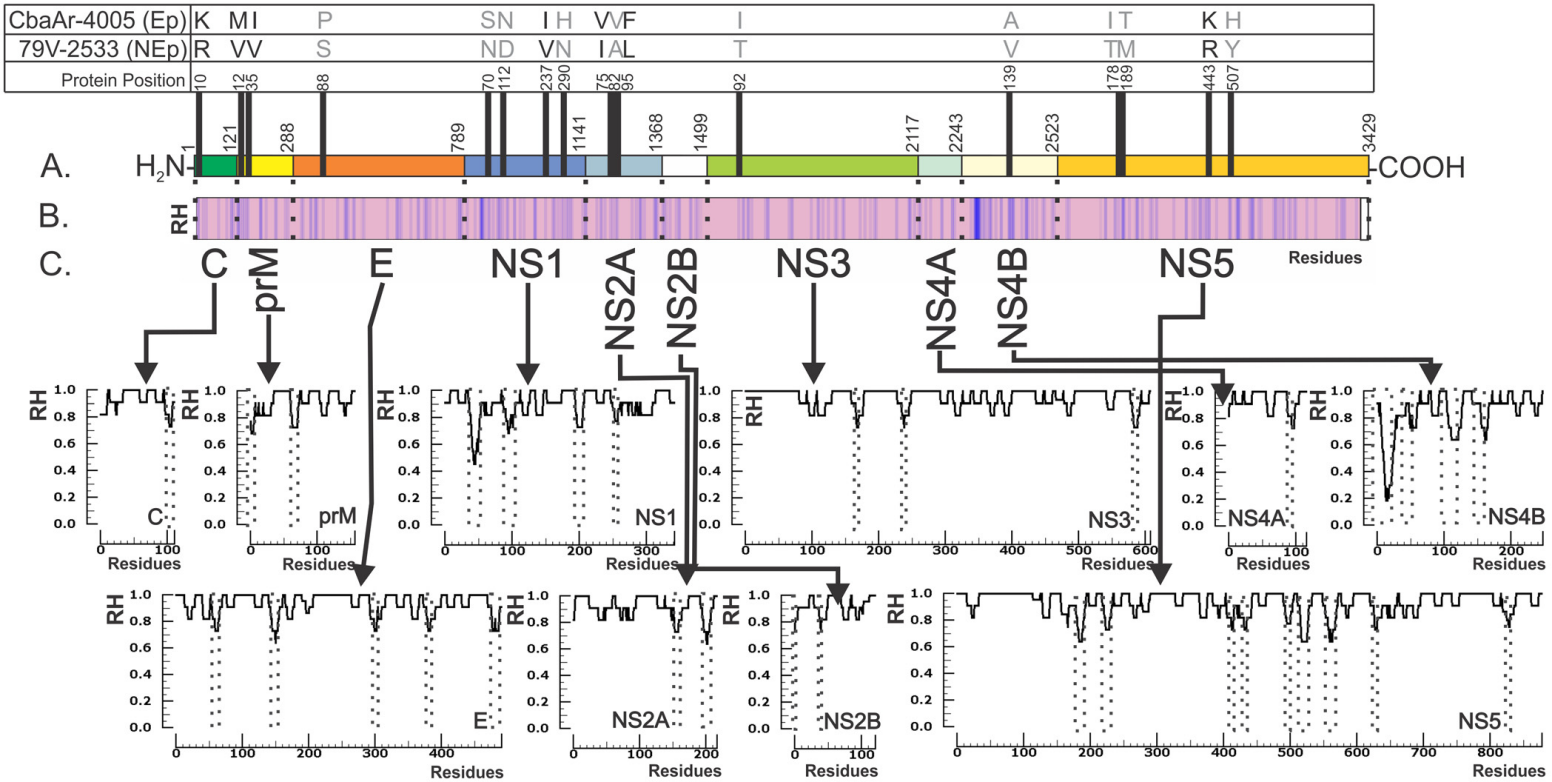
Amino acid differences between epidemic (Ep) and non-epidemic (NEp) SLEV strains and the polyprotein conservation profile. The horizontal bar represents the SLEV polyprotein. (**A).** Black vertical lines represent residues (in one letter code) that differed between strains studied (CbaAr-4005 (Ep) and 79V-2533 (NEp)). In grey, the non-conservative changes and their respective individual protein positions are highlighted. (**B).** Polyprotein homology profile for all studied SLEV strains. The light pink areas show the more conserved regions while in the less conserved zones are in blue. (**C).** Individual protein homology profile for all studied SLEV strains. The Y axis shows the Relative Homology (RH) between 0 and 1 while the X axis shows the residue position. In each protein profile, the zones that show an RH value lower than 0.8 are boxed.

Full length, consensus genome sequences from both SLEV strains examined (CbaAr-4005 and 79V-2533) were obtained using traditional RT-PCR and capillary sequencing. Each full length genome consisted of a total of 10936 nt. The ORF is composed of 3429 amino acids (10287 nt) while the 5′and 3′untranslated regions (5′UTR and 3′UTR) consisted of 98 nt and 548 nt, respectively. When comparing the Ep vs NEp SLEV strains, a total of 133 changes were observed at the nucleotide level resulting in17 amino acids substitutions (13% of the total amino acids). Of these 17, 10 non-conservative changes were found (59%) (Fig 1). Only four changes in the structural proteins were identified, with only one being non conservative (E_88_) (Table 1). The remaining amino acid substitutions were identified in nonstructural proteins. Of these changes, 9 were considered non conservative: NS1_70_, NS1_112_, NS1_290_, NS2A_82_, NS3_92_, NS4B_139_, NS5_178_, NS5_189_, NS5_507_ (Table 2).

**Table 2:** Conservative and non-conservative amino acids mutations detected between SLEV strains CbaAr-4005 and 79V-2533.

Protein_/position_	Viral strain		Dayhoff classification criteria
	CbaAr-4005(Epidemic)	79V-2533(Non-Epidemic)	
C_10_	K	R	Conservative
prM_12_	M	V	Conservative
prM_35_	I	V	Conservative
E_88_	P	S	Non-conservative
NS1_70_	S_Ep_	N_NEp_	Non-conservative
NS1_112_	N_Ep_	D_NEp_	Non-conservative
NS1_237_	I_Ep_	V_NEp_	Conservative
NS1_290_	H_Ep_	N_NEp_	Non-conservative
NS2A_75_	V_Ep_	I_NEp_	Conservative
NS2A_82_	V_Ep_	A_NEp_	Non-conservative
NS2A_95_	F_Ep_	L_NEp_	Conservative
NS3_92_	I_Ep_	T_NEp_	Non-conservative
NS4B_139_	A_Ep_	V_NEp_	Non-conservative
NS5_178_	A_Ep_	V_NEp_	Non-conservative
NS5_189_	I_Ep_	T_NEp_	Non-conservative
NS5_443_	K_Ep_	R_NEp_	Conservative
NS5_507_	H_Ep_	Y_NEp_	Non-conservative

The relative homology profile carried out with all 27 SLEV strains analyzed indicated that amino acid mutations occurred along the entire genome (without inclusion of the Palenque strains). Hypervariable regions (HR ≤ 0.6) were detected only in NS1 and NS4B (Fig 1), while variable regions were present in the rest of the proteins. Most of the compared sequences showed indices ≥ 0.8, with few regions between 0.6 and 0.8, indicating high conservation among SLEV strains. When Palenque strains were included the appearance of some areas with more variability in NS3 and NS5 proteins were observed.

### Protease cleavage sites

The identified cleavage sites remained preserved in most of the SLEV strains analyzed. Exceptions include the BeAn-247377 strain where the standard SWPAS site between NS2A and NS2B was found to be GWPAS, and LALGM between NS3 and NS4A changed to AAGKR in strain I MP115. Differences in amino acids at positions 2, 4, and 5 relative to the cleavage site were also detected. Less conservation can be found in the C protein of the Palenque strains where the conserved motif **PSKKR/GGTRS** changed to **PSKK** **K** **/GG** **SG** **S**. The protease involved in this cleavage is of viral origin, suggesting this change may be related with changes in the catalytic site of the enzyme by modifying its recognition site. The cleavage sites located between NS4A/2K remain conserved among the flaviviruses analyzed (ROCV, WNV, JEV, MVEV); while most other sites exhibited some degree of variation at amino acids 1 and 2 (Table 3).

**Table 3:** Protease cleavage sites identified in SLEV sequences analyzed.

Cleavage Site	Protease	SLEV	Changes (Strains)
**VirC/AnchC**	viral serine protease	**PSKKR/GGTRS**	**PSKKR/GGT** **G** **S**(ImperialValley, 79V-2533, CbaAr-4005, CorAn9124, CorAn9275, BeAn247377, GML902612, GML903797, TRVL9464, 75D90, BeAn246262, BeAr23379)
			**PSKKR/GGTR** **P**(GMO94)
**AnchC/prM**	host signalase	**GLASS/LQLST**	**GLASS/LQLS** **N**(69M1143)
**Pr/M**	furin	**RRSRR/SISVQ**	**K** **RSRR/SISVQ**(TRVL9464)
**M/E**	host signalase	**APAYS/FNCLG**	-
**E/NS1**	host signalase	**TSVQA/DSGCA**	-
**NS1/NS2A**	Unknown	**SRVTA/GVAGG**	**SRVTA/G** **I** **AGG** (69M1143)
			**SRVTA/GVGG** (Parton)
**NS2A/ NS2B**	viral serine protease	**PNGKR/SWPAS**	**PNGKR/** **G** **WPAS** (BeAn247377)
			**PNGKR/SWPA** **G**(72V-4749)
**NS2B/NS3**	viral serine protease	**KHSKR/GGALW**	-
**NS3/NS4A**	viral serine protease	**AAGKR/SALGM**	**AAGKR/** **L** **ALGM** (Imperial Valley)
**NS4A/2K**	viral serine protease	**EPEKQR/SQTDN**	-
**2K/NS4B**	host signalase	**GVVAA/NEMGL**	**GVVAA/NEM** **D** **L** (Hubbard)
**NS4B/NS5**	viral serine protease	**PKGKR/GGGKG**	-

### Secondary structure and signals in non-coding regions

Only a few changes were detected between Ep and NEp strains in the non-coding sequences. A total of 26 transitions and 3 transversions (1 for the 5’UTR and 2 for the 3’UTR) were identified. For SLEV, only three strains have been sequenced in these regions: Kern217, Hubbard, and Imperial Valley. When comparing available sequences with those reported here (CbaAr-4005 and 79V-2533), only a few changes were observed; the changes are mainly transitions as only 11.5% of the changes were transversions and only one INDEL was present.

**Fig 2:**
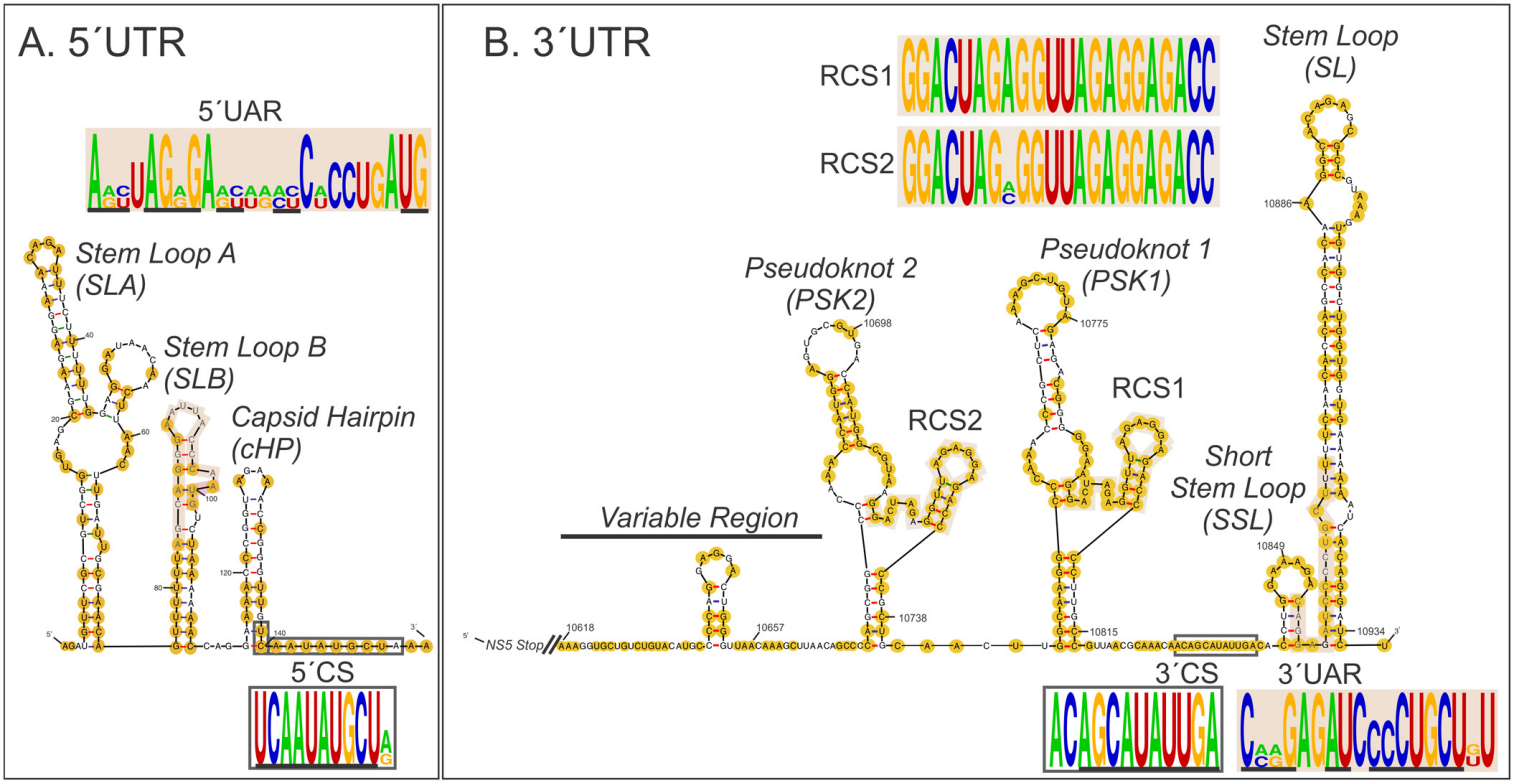
Predicted secondary structures for 5′UTR and 3′UTR of a NEp SLEV strain. For both ends, the conserved sequences (grey shadowed and boxed) and structures being comparison with DENV2 are shown [16]. The conserved residues in the alignment are shadowed. (**A).** 5′UTR scheme, where structures of Stem Loop A (SLA), Stem Loop B (SLB), capsid hairpin (cHP), and the 5′ upstream AUG region (5′UAR), and 5′ conserved sequence (5′CS) are shown. (**B).** 3′UTR scheme, showing the Stem Loop (SL), Short Stem Loop (SSL), the Pseudoknot 1 and 2 (PSK1 and PSK2), Variable Region, the 3′ upstream AUG region (3′UAR) sequences, the 3′ conserved sequence (3′CS), and the repeated conserved sequences 1 and 2 (RCS1 and RCS2).

The predicted structure was made using MFold with the sequence data from strain 79V-25533;the results are shown in Fig 2. For both Ep and NEp strains, the same structures were obtained. For the 5′UTR and 3′UTR ends, both stem loop structures and pseudoknots were identified. For the 5′UTR, there are three stem loop structures (SLA, SLB, and capsid hairpin–cHP-), while for the 3′UTR, one large stem loop (SL) and one short stem loop (SSL) were found close to the 3′ end followed by two pseudoknot structures (PK1 and PK2) and a third stem loop.

Fig 2 shows the conserved structures identified in non-coding regions for the 2 SLEV strains sequenced and 2 sequences of DENV2 (NC001474.2: DENV-2/16681 and DQ181806.1: DENV-2/Th2_0038_74). The 5′ upstream UAG region (5′UAR) and 5′ conserved sequence (5′CS) in the 5′UTR (Fig 2A), and the 3′ upstream UAG region (3′UAR), the 3′ conserved sequence (3′CS) and repeated conserved sequence 1 and 2 (RCS1 and RCS2) in the 3′UTR (Fig 2B) were highlighted. The Sequence Logos from alignment of the respective portions are shown.

### Pathogenicity and Viremia phenotypes

**Fig 3:**
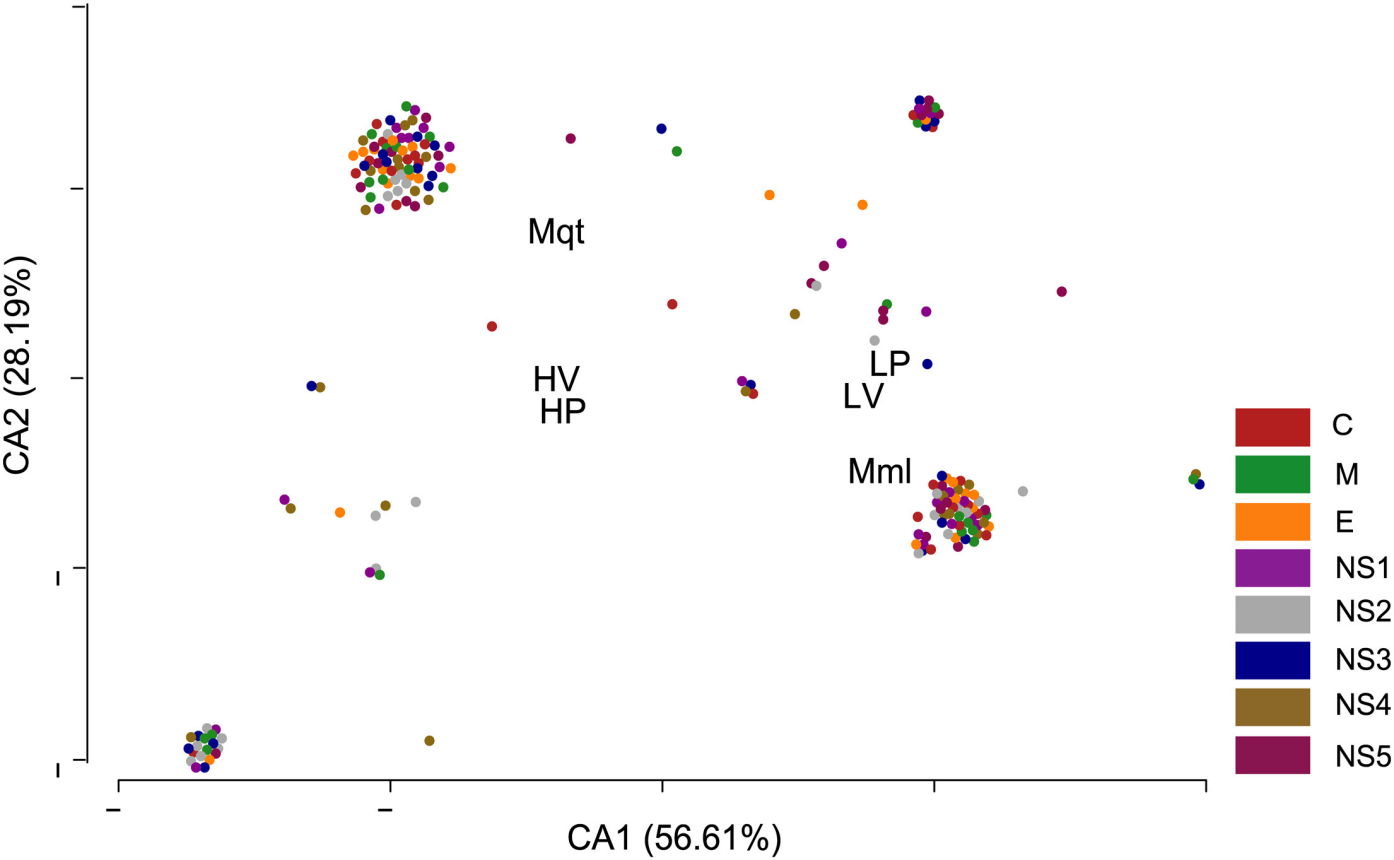
Correspondence analysis exploring the distribution of 200 non-conservative mutations in 14 SLEV strains through biological features. Axes CA1 and CA2 account for 84.8% of the total variation observed. HP: high pathogenicity, LP: low pathogenicity, HV: high viremia, LV: low viremia. Source of isolation: Mqt: mosquitoes, Mml: mammals, Brd: birds. Colored dots represent each mutation analyzed distributed by protein.

**Fig 4:**
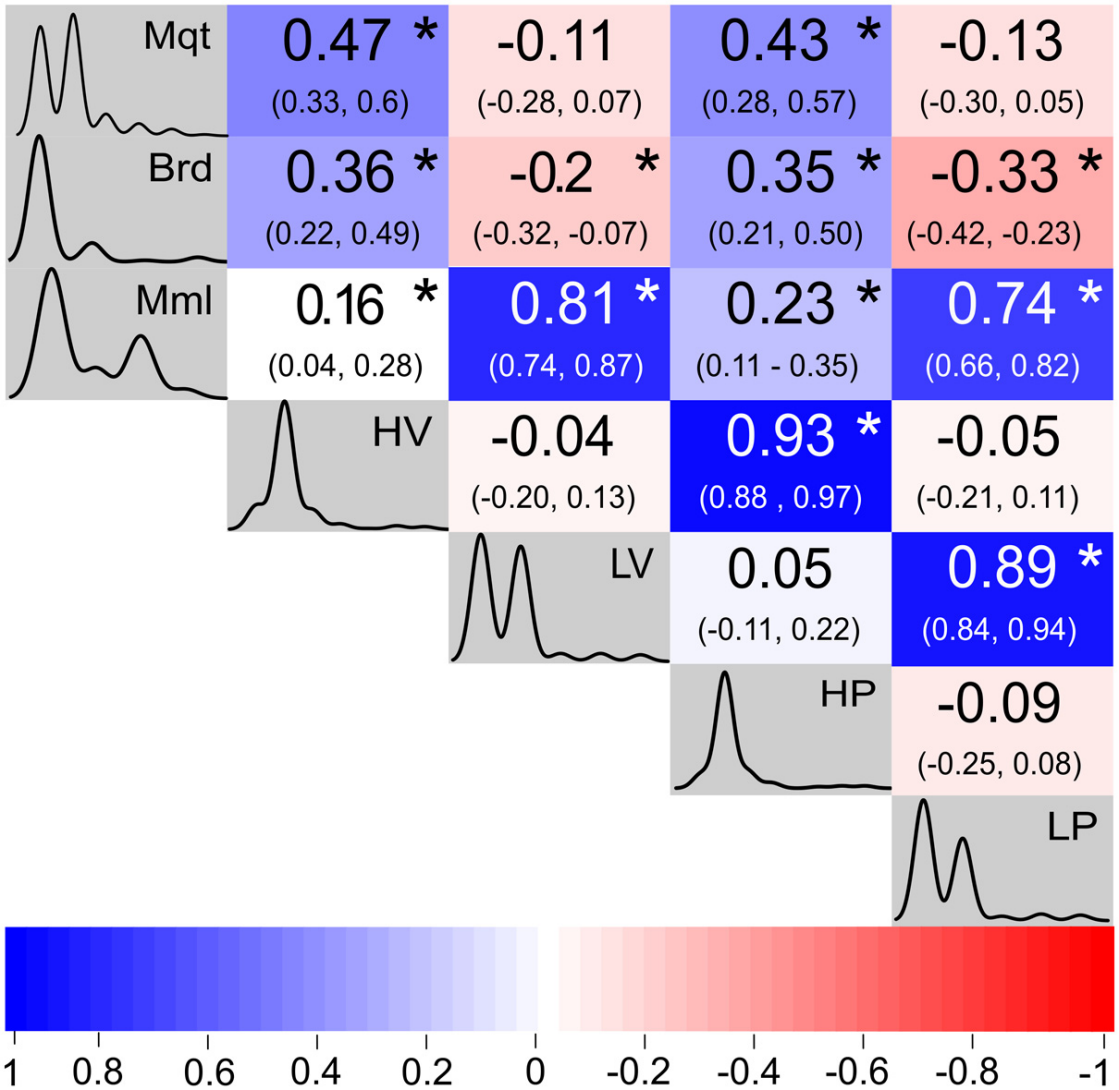
Spearman’s correlation indices and confidence intervals obtained comparing source of isolation (Mml: mammals, Brd: birds, Mqt: mosquito) and biological behavior (viremia in birds and pathogenicity in mice)in 14 SLEV strains. Gradient scale colors indicate strength of correlation (positive or negative). *: statistically significant differences.

A total of 200 non-conservative mutations were mapped across 14 SLEV strains. Their association with biological features was investigated. According to the CA, the first two axes account for 84.8% of the total variation observed in the data set (Fig 3). High correlations between HV-HP and LV-LP mutations were observed. This pattern was confirmed through the Spearmans’ correlation test, where strains that developed high viremia in birds were also found to be highly pathogenic in mice (see Fig 4 for Spearman’s correlation index values). Interestingly positive and significant correlations were detected among strains isolated from Mml/LP and Mml/LV. Strains isolated from mosquitoes showed significant correlations with those HV and HP strains (Fig 4). However, no statistically significant differences were observed between specific point mutations and their presence in high/low viremia strains and high/low pathogenic strains (Exact Fisher’s test; p>0.01).

### Phylogenetic analyses

**Fig 5:**
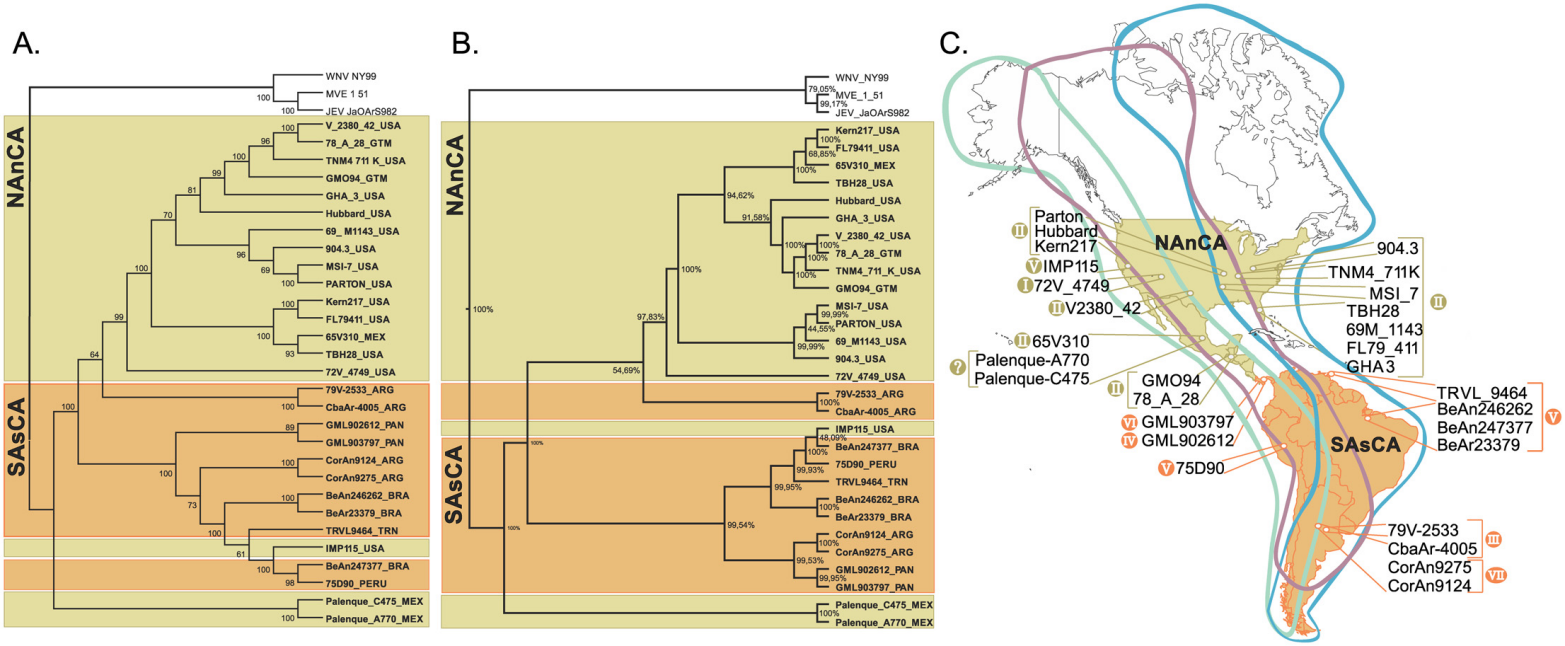
Phylogenetic analyses performed using the SLEV sequences listed in Table 1. (A): Maximum Likelihood analysis. (B): Bayesian inference analysis. (C) Geographic distribution of analyzed St. Louis encephalitis virus strains and migratory bird flyway. West Nile virus (Acc. Number: DQ211652), Murray Valley Encephalitis virus (Acc. Number: AF161266) and Japanese Encephalitis virus (Acc. Number: M18370.1) were used as outgroups. Abbreviations for the different geographic origin of strains were used as follow: USA (United States); MEX (Mexico); GTM (Guatemala); ARG (Argentina); BRA (Brazil); PERU (Peru); TRN (Trinidad); PAN (Panamá).

Both Maximum Likelihood (Fig 5A) and Bayesian analyses (Fig 5B) methods showed the same phylogenetic reconstruction. The best fit model for both methods was GTR+I+G (parameters: G = 1, I = 0.38). High confidence values per node were observed with bootstrap values ranging from 70–100. The tree presented (Fig 5B) is the maximum clade credibility tree summarized from MCMC data with a burn in of 10%.

No pattern was found among phenotype (high/low viremia in birds, high/low pathogenicity in mice, or source of isolation) and phylogenetic classification. Additionally, no patterns based on either the host or year of isolation were identified. However, two main groups reflecting geographic origin could be distinguished: North America/northern-Central America (NAnCA) (Genotypes I and II: USA, MEX and GTM) and South America/southern-Central America (SAsCA) (Genotypes III, IV, V, VI and VII: ARG, BRA, PERU, PAN and TRN) (Fig 5C). Occasional exceptions were found such as one strain from USA (Imperial Valley, CA) that clustered in the SAsCA group. Although CbaAr-4005 and 79V-2533 strains were close to the NAnCA cluster, they constituted a separate lineage (Fig 5). In order to corroborate this result, a Procrustes analysis was carried out. It showed a high correlation among geographic and genetic distance. The goodness of fit in the NMDS was 0.012 and 0.039 for geographic and genetic distances, respectively. The configuration obtained between NMDSs based on 10000 permutations was highly significant (*t0 = 0*.*521*, *m* ^*2*^ *= 0*.*728 scale factor = 0*.*521*, *p-value = 0*.*001*).

## Discussion

The occurrence of human encephalitis outbreaks in Central and South America by flaviviruses is historically unusual. The first and largest encephalitis outbreak in human populations reported in South America was caused by *Rocío virus* in coastal region of southern Sao Paulo, Brazil. During that outbreak, a total of 465 cases with 61 deaths was recorded[27]. Beginning in 2002, human encephalitis caused by SLEV became frequent in Argentina and Brazil, with extra heamorrhagic manifestations reported in patients from Brazil [3]. Human outbreaks in populated areas of Argentina were reported in Córdoba (2005), Paraná (2006), Buenos Aires (2010), and San Juan (2012)[1,5,28]. Molecular evidence suggested some association between genotype III SLEV strains and encephalitis[9,10]. In this study, we explored potential molecular markers with epidemiological and biological features through *in silico* sequence comparison and bioinformatic analysis of epidemic and non epidemic SLEV strains isolated in the Americas.

The substitutions identified in the flavivirus structural proteins (C, prM and E), nonstructural proteins (NS1, NS2A, NS2B, NS3, NS4A, NS4B and NS5) and the 3′ and 5′ UTRs influence the biological (virulence, neuroinvasiveness, and viremia profiles) and adaptive behavior of the viruses[29,30]. In our study comparing the coding region of Ep and NEp SLEV strains, we detected 17 amino acid changes of which 59% were non-conservative (Fig 1). Some of these mutations are responsible for differences in biological behavior observed between Ep and NEp SLEV strains in animal models[12,23–25].

This study explored the association between 200 non-conservative point mutations and biological characteristics. Although multivariate analysis suggested there were associations of pooled mutations with biological features (Fig 3), no statistically significant differences were detected when analyzing mutations individually. This could be explained by the low number of viral strains characterized. Additionally, the difficulty in identifying molecular markers related to particular phenotypic characteristics could be masked or lost by compensatory sequence mutations that modify the viral fitness when the strains are subjected to cell culture for isolation[31,32].

It was also observed that several strains showed changes at cleavage site residues but only when the viral serine protease is involved in the processing at that site (Table 3). In the P1 and P2 positions, a predominance of positively charged amino acid residues were observed, while a glycine residue (G) is present in positions P1′ and P2′ (site P2P1/P1′P2′), in both DENV and WNV. It was also observed in both viruses, that an Arg or Lys was absolutely necessary for the cleavage at P1 or P2, while the Gly presence was not exclusively required for DENV [33]. Likewise, Arg or Lys residues were present in every strain of SLEV analyzed here. While WNV and DENV had a G residue at P1’ and P2’, a Lys was commonly found at P2’ in the SLEV strains. A reverse genetic system would be necessary to analyze these changes and determine their impact on the phenotype.

Of particular interest is the strong correlation detected between viremia in birds and pathogenicity in mice. It is known that SLEV viral strains are not able to cause neurological damage in birds but they do develop high viremias. This finding is contrary to what is observed during SLEV infection in mice, where viral loads are low but neurological signs are observed. Our findings suggest that mutations important for viral replication in birds are also significant in the neuroinvasion process in mammals. Experimental evidences suggests a role for E and non-structural proteins during this process [34].

Two reports described the conserved UTR secondary structure of SLEV and other flaviviruses[35,36]. Because these structures are highly conserved among flaviviruses, it is possible to infer their functional importance in viral RNA replication [37] and translation[38]. For example, it has been reported that changes in the WNV 3′UTR (along with other mutations in the coding region) generate temperature sensitive phenotypes, reduce plaque production, and result in attenuated neurovirulence in rodents[39]. A high degree of sequence conservation was observed when comparing DENV-2 and SLEV strains (Fig 2). For the potential 5′UAR (5′-AGCAGGGAAUUACCCAAUG-3′) found in the 5′UTR, complementary residues (underlined) within the 3′UAR (5′-CAGGAGAUCCCCUGCUUU-3′) were identified. These regions likely come together to form the panhandle structure for circularization of the genomic RNA such as is observed for other flaviviruses[40]. The RCS1 and RCS2 are totally conserved between these viruses and are present in all mosquito-borne flaviviruses. They have been shown in DENV to be implicated in RNA synthesis [41]. However, the impact of subtle variations in these regions between strains is unknown and therefore, it is very important to undertake studies assessing the structure and function of these regions in SLEV strains with different phenotypes.

The phylogenetic analysis based on complete ORF data and Procrustes analysis indicates a strong association between SLEV strains with their geographic distribution with two main clades based on geography (North America /northern Central America and South America/southern Central America) (Fig 5). However, some exceptions are observed indicating that viral flow among the SLEV strains across its geographic range does occur [42]. Because SLEV is maintained by mosquito vectors and avian hosts, the actual distribution of SLEV along its northward and southward dispersion could be explained by bird migration. The same dispersal mechanism was postulated for WNV and its distribution across the Americas [43]. Although not completely understood, birds migrate across the continent through 3 main flyways (Pacific Americas, Mississippi Americas and Atlantic Americas flyways) (Fig 5C). Interestingly, the presence of South American genotype V SLEV strains in North America has been reported in California, Florida and Texas[8] overlapping with migration flyways (Fig 5C). Conversely, North America genotype II was reported in Argentina and Brazil[8,11]. Based on previously available genetic data, Auguste et al.[42] suggested that SLEV may have originated in Brazil. However, recent evidence obtained from the isolation of a new SLEV variant (Palenque strain) indicates a Central America origin[44].

This study represents the first theoretical step in understanding the molecular mechanisms underlying the virulence features and biological variation among SLEV strains. We have identified highly variable sites and point mutations among high pathogenicity/high viremia and low pathogenicity/low viremia strains that may provide the foundation necessary for development of molecular biology tools such as reverse genetic systems for SLEV. These tools will allow us to identify molecular markers for SLEV virulence and to dissect the mechanisms driving these interactions.

## Supporting Information

S1 File Procrustes analysis script to run on R. (PDF)
