# Supplementary material for: Exploring Genomic, Geographic and Virulence Interactions among Epidemic and Non-Epidemic St. Louis Encephalitis Virus (Flavivirus) Strains
Source: PLoS One. 2015 Aug 27;10(8):e0136316. doi: 10.1371/journal.pone.0136316 (PMC4552378; doi:10.1371/journal.pone.0136316)
Supplement: S1 File — (PDF) [file pone.0136316.s001.pdf]

```
####### Procrustes analysis relation between geographic and genetic distance of SLEV strains

library(vegan)

geo.slv<-read.csv("slv_geo.csv",dec=".")
genslv<-read.csv("slv_gen.csv",sep=";",dec=",",header=FALSE)

#Remove outliers

genslv<-genslv[-c(6:7),]
genslv<-genslv[,-c(6:7)]
mgenslv<-as.matrix(genslv)
geo<-geo.slv[-c(6:7),-1]

####Geodesic distance matrix between isolates sites of SLEV strain

geodetic.distance.dataframe <- function(dataframe, lat, long) {
      NSITES <- nrow(dataframe)
      latitude <- dataframe$lat
      longitude <- dataframe$long
      latlong <- cbind(latitude, longitude)*pi/180
      d <- matrix(nrow=NSITES, ncol=NSITES)
      for(i in 1:(NSITES-1)) {
      d[i,i] <- 1.0
           for (j in (i+1):NSITES) {
                 d[i,j] <- sin(latlong[i,1])*sin(latlong[j,1]) + cos(latlong[i,1]) *
cos(latlong[j,1])*cos(abs(latlong[i,2] - latlong[j,2]))
                 d[j,i] <- d[i,j]
           }
      }

d[NSITES, NSITES] <- 1.0
d <- acos(d)

for (i in 1: NSITES) {
   for (j in i:NSITES) {
     if (d[i,j] < 0.00000000001) {d[i,j] <- 0}
     d[j,i] <- d[i,j]
         }
      }
d <- ifelse (d < 0.000000000001, 0.0, d)
R <- 6371
d <- R*d
d
}


geo.dis<-geodetic.distance.dataframe(geo)
geo.dis

###NMDS of genetic distance between strains of SLEV

Mgen<-metaMDS(vegdist(mgenslv),autotrasnform=F,trace=F,trymax=1000,k=2)
```

```
plot(Mgen)
Mgen.stress<-stressplot(Mgen,vegdist(mgenslv), main="NMDS SLEV Genotypes")

###NMDS of geographic distance between sites of SLEV strain isolation

Mgeo<-metaMDS(vegdist(geo.dis),k=2,autotrasnform=F,trace=F)
Mgeo.stress<-stressplot(Mgeo,vegdist(geo.dis), main="NMDS SLEV Geo")
ordiplot(Mgeo,type="t")


#### Procrustes

pro.pro<-procrustes(Mgeo,Mgen,scores="sites",scale=T,symmetric=T,permutation=10000)

postscript(file="biplot_slevGeoGen1",paper="a4",horiz=T)
plot(pro.slv4,main=NA,ylab=NA,xlab=NA)
text(-0.35,0.3, cex=.8,"R rotation=0.3")
text(-0.35,0.25, cex=.8,"p-value=0.108")
text(-0.35,0.20, cex=.8,"Scaling=0.03")
dev.off()

plot(pro.pro,kind=2)

####Permutation test of Procrustes analysis

pro.slv4<-protest(Mgen,Mgeo,scores="sites",scale=T,symmetric=T,permutation=10000)

tO<-pro.slv4$t0
angulo<-pro.slv4$Yrot
tp<-pro.slv4$t
pro.slv4$signif
pro.slv4$rotation
pro.slv4$translation
pro.slv4$scale
pro.slv4$ss
residuals(pro.slv4)
summary(pro.slv4)

postscript(file="permutation_slevGeoGen",width=7,height=6, paper="a4",horiz=T)
hist(tp,main=NA, ylim=c(0,2500))
abline(v=tO, col="red")
text(.4,2400, cex=.8,"p-value=0.001")
text(.37,2200, cex=.8,"t0=0.521")
dev.off()

plot(pro.slv4,kind=2,main=NULL)
```
